# The Prognostic Value of Tumor-infiltrating Lymphocytes in Hepatocellular Carcinoma: a Systematic Review and Meta-analysis

**DOI:** 10.1038/s41598-017-08128-1

**Published:** 2017-08-08

**Authors:** Wei Yao, Jun-chuang He, Yan Yang, Jian-ming Wang, Ya-wei Qian, Tao Yang, Lei Ji

**Affiliations:** 1 Department of Biliary and Pancreatic Surgery/Cancer Research Center Affiliated Tongji Hospital, Tongji Medical College, Huazhong University of Science and Technology, Wuhan, Hubei 430030 China; 2 Department of Hepatobiliary and Pancreatic Surgery, Henan Provincial People’s Hospital, Zhengzhou, Henan 450003 China; 3 Department of Hepatobiliary Pancreatic Surgery, Affiliated Hospital of Hainan Medical University, Haikou, Hainan 570102 China

## Abstract

Previous clinical studies have found that the levels of tumor-infiltrating lymphocytes (TILs) significantly correlated with prognosis in hepatocellular carcinoma (HCC). However, these conclusions and data remain controversial. We performed a systematic review and meta-analysis to assess the prognostic value and clinical utilization of TILs in patients with HCC. A total of 23 relevant studies of 3173 patients were included into our meta-analysis. The results demonstrated that high levels of CD8^**+**^ and CD3^**+**^ TILs had a better prognostic value on overall survival (OS), with HRs of 0.71 (*P* = 0.04) and 0.63 (*P* = 0.03), respectively, compared to low levels, as did high levels of CD8^**+**^, CD3^**+**^ and CD4^**+**^ TILs on disease/recurrence-free survival (DFS/RFS), with HRs of 0.66 (*P* = 0.01), 0.60 (*P* = 0.01) and 0.79 (*P* = 0.04), respectively. In contrast, high levels of FoxP3^**+**^ TILs had a worse prognostic value on OS and DFS/RFS, with HRs of 2.06 (*P* < 0.00001) and 1.77 (*P* < 0.00001), respectively. The FoxP3^+^/CD4^+^ and FoxP3^+^/CD8^+^ ratios negatively correlated with OS and DFS/RFS. These findings suggest that TILs may serve as a prognostic biomarker in HCC. However, further research should be performed to clarify the clinical value of TILs in HCC.

## Introduction

The oncogenesis and development of malignant tumors, including initiation, progression, malignant conversion, invasion and metastasis, are dynamic processes that involve multiple links, stages and genes. Traditional cancer research exclusively focuses on the internal changes of tumor cells themselves, including genetic and phenotypic changes. However, the continuous progress of gene and molecular biology technology has revealed the complicated functions of the tumor microenvironment in tumor evolution^1–3^. The tumor microenvironment plays a vital role in tumor epigenetics, tumor differentiation, immune escape, and infiltration metastasis. The host immune response and immune cells are crucial factors of the tumor microenvironment that are consistently involved throughout tumor development^4^. The immune response has a vital function via regulation of carcinogenesis and cancer progression, including promotion and suppression^5, 6^. Related research has demonstrated that immune factors are accurate independent prognostic factors that are superior to the TNM stage^7, 8^. Cancer immunologists and cancer biologists achieved a consensus that cancer is a disease of the microenvironment and immunity^9^. Current research has also demonstrated that immunotherapy plays a valuable role in anti-tumor treatments, such as active vaccination, adoptive cell transfer therapy and immune checkpoint blockade. Various therapies are being assessed in clinical trials, and the results have demonstrated a definite clinical application value^10^. Therefore, tumor-infiltrating lymphocytes (TILs), as the most important monitor of the immune response, are a focus of cancer research.

TILs are a group of lymphocytes located around tumor cells that exhibit diverse functions in various subsets. TILs have been identified in primary tumors, lymph nodes, and metastases. CD3^+^, CD4^+^, CD8^+^ and FoxP3^+^ T lymphocytes are the most common subsets of TILs. CD8^+^ T lymphocytes primarily belong to cytotoxic T lymphocytes (CTLs), which are primarily responsible for the removal of target cells, including tumor cells. CD4^+^ T lymphocytes, which are also known as the “auxiliary hand” of the immune system, are referred to as T helper lymphocytes (Ths). Mosmann *et al*.^11^ first divided CD4^+^ T lymphocytes into Th1 and Th2 cells in the early 1980s based on different cell functions and cytokines secreted. Th1 cells enhance the toxic effects of killer cells, such as activating CTLs, or stimulate a delayed-type hypersensitivity to mediate the cell immune response. Th2 cells promote antibody production and mediate the humoral immune response. Researchers also confirmed that other subsets exist in CD4^+^ T lymphocytes, such as CD4^+^ regulatory T lymphocytes (Tregs), which characteristically express Forkhead box P3 (Foxp3). Tregs are the most important immunosuppressive cells in the body^12, 13^. The ratios of the different subsets also have important implications in carcinogenesis. The value of TILs in oncology is not difficult to imagine based on the important position of these cells in tumor immunity, and immune cells, especially TILs, have been a hotspot in cancer research. TILs may present a key breakthrough for anti-tumor therapy.

HCC is one of the most common cancers worldwide, and it has attracted widespread attention because of its high incidence and mortality rate^14^. The prognosis of HCC patients remains dismal despite the enormous achievements made in clinical treatments during recent decades. There is an urgent need for related targeted molecules to predict outcomes and for use as oncotherapy in HCC. Extensive research has assessed the relationship between TIL levels and HCC, particularly tumor characteristics and prognostic outcome. Some conclusions have been mentioned previously, but the results remain inconsistent and debatable in HCC. We performed a meta-analysis based on data acquired from published studies using specific inclusion and exclusion criteria to clarify the prognostic value of TILs and the ratios of different subsets in HCC. Hazard ratios (HRs) and 95% confidence intervals (95% CIs) were used as effect measures.

## Results

### Study selection and characteristics

The full texts of 91 articles were scrutinized. Twenty-nine of these articles did not report adequate data to calculate HRs and 95% CIs, and 16 articles were studies of peri-tumoral tissues or peripheral blood. Nine articles were not related to survival analyses, and 7 articles were categorized as meta-analyses, review articles, or case reports. Seven articles were non-English reports. All of these articles were excluded. We identified 23 articles for inclusion in this meta-analysis^15–37^. Our search and selection processes were performed in strict adherence with the inclusion and exclusion criteria.

These observational retrospective studies evaluated TIL levels and prognostic parameters in HCC from 2004 to 2016. Six of the eligible studies assessed CD3^+^ T lymphocytes, and 4 studies investigated CD4^+^ T lymphocytes. Fourteen studies examined CD8^+^ T lymphocytes, and 13 studies reported FoxP3^+^ T lymphocytes. Only 2 studies reported FoxP3^+^/CD8^+^ ratios, and 3 studies reported FoxP3^+^/CD4^+^ ratios. Two studies assessed CD8^+^/CD3^+^ ratios. Overall survival (OS) and disease-free survival (DFS) were commonly assessed. However, recurrence-free survival (RFS) and cancer-related/specific survival (CRS/CSS) were not generally reported. CRS/CSS was only reported for FoxP3^+^ T lymphocyte levels. The median number of patients evaluated per study was 138, and 4 studies included more than 200 patients. Most studies were from Asia, especially China. The clinicopathological characteristics of the patients involved generally provided information on HBV infection, liver cirrhosis, TNM-stage, Child-Pugh score, tumor number, and vascular invasion. Table 1 summarizes some of the characteristics of the eligible studies in the present systematic review and meta-analysis. And Newcastle-Ottawa Scale (NOS) scores of the studies ranged from 5 to 8.

**Table 1:** Characteristics of the involved studies.

Study (reference)	Ethnicity	No.of patients (Male, %)	Age, median (range) or mean, y	HBsAg (+) (%)	liver cirrhosis (%)	Child-pugh A (%)	TNM stage I–II (%)	Multiple tumor (%)	Vascular invasion (%)	TILs subsets	Outcomes	NOS
Wang 2016^15^	Asia/non-Asia	66 (80.0%)	55.4 ± 11.0	NR	100%	NR	56.0%	NR	85.0%	CD8^+^,Foxp3^+^	OS,RFS	5
Gabrielson 2016^16^	Asia/non-Asia	65 (76.92%)	61 (30–86)	39.23%	35.38%	NR	76.92%	NR	32.31%	CD3+,CD8+	OS,RFS	5
Sun 2015^17^	Asia	359 (88.6%)	50 ± 13.7	91.2%	49.6%	NR	57.5%	25.5%	NR	CD3+, CD8+	OS,DFS	6
Brunner 2015^18^	Europe	119 (78%)	65 (58–71)	5%	60%	58%	69%	NR	NR	CD8^+^	OS	7
Garnelo 2015^19^	Asia/Europe	103 (NR)	NR	NR	NR	NR	NR	NR	NR	CD3^+^	OS	5
Lin 2013^20^ ^a^	Asia											6
Training cohort		132 (92%)	51 (20–71)	82%	92%	67%	62%	48%	27%	CD3^+^,CD4^+^, CD8^+^,Foxp3^+^, Foxp3^+^/CD4^+^, CD8^+^/CD3^+^	OS,DFS	6
Validation cohort		113 (80%)	52 (32–73)	84%	91%	68%	63%	34%	26%	CD3^+^,CD4^+^, CD8^+^,Foxp3^+^, Foxp3^+^/CD4^+^, CD8^+^/CD3^+^	OS,DFS	6
Chen 2012^21^	Asia	141 (87.9%)	51.7 ± 9.8	82.3%	76.6%	82.3%	67.4%	40.4%	74.5%	CD3^+^,CD4^+^, CD8^+^,Foxp3^+^, Foxp3^+^/CD4^+^, CD8^+^/CD3^+^	OS,DFS	6
Mathai 2012^22^	USA	91 (79.1%)	56 (25–81)	69.2%	NR	NR	91.2%	NR	22%	Foxp3^+^/CD8^+^	OS,DFS	8
Huang 2012^23^	Asia	54 (83.3%)	53.2 ± 11.6	79.6%	87.3%	74.1%	42.6%	27.8%	64.8%	Foxp3^+^, Foxp3^+^/CD8^+^	OS,DFS	7
Gao 2012^24^	Asia	206 (90.3%)	49 (24–72)	93.2%	95.1%	60.7%	61.7%	60.2%	57.8%	CD8^+^,Foxp3^+^	RFS,CSS	5
Wang 2012^25^	Asia	137 (81.8%)	56 ± 10	38.7%	89.1%	69.3%	NR	30.7%	NR	CD8^+^,Foxp3^+^	OS,RFS	5
Li 2011^26^	Asia	197 (83.3%)	53 (18–81)	93.4%	88.3%	100%	88.8%	20.8%	35.5%	CD8^+^	OS,RFS	6
Chen 2011^27^	Asia	143 (87.4%)	52 ± 10.1	83.9%	73.4%	81.1%	66.4%	38.5%	NR	Foxp3^+^	OS	5
Shen 2011^28^	Asia	76 (92.1%)	NR	92.1%	81.6%	97.4%	NR	28.9%	NR	Foxp3^+^	OS,DFS	7
Lin 2010^29^	Asia	102 (83.3%)	49.5 (13–75)	NR	76.5%	NR	73.5%	NR	NR	Foxp3^+^	OS	7
Zhou 2009^30^	Asia	85 (NR)	NR	NR	NR	NR	NR	NR	NR	Foxp3^+^	OS,DFS	6
Gao 2009^31^	Asia	240 (85%)	52 (18–81)	92.9%	88.3%	99.6%	75.8%	23.3%	45.4%	Foxp3^+^	OS,DFS, CSS	6
Pang 2009^32^	Asia	12 (83.3%)	NR	NR	NR	NR	66.7%	NR	NR	CD4^+^,CD8^+^	OS	5
Sasaki 2008^33^	Asia	164 (76.8%)	63.9	22.6%	51.2%	NR	NR	NR	NR	Foxp3^+^	DFS,CRS	6
Gao 2007^34^	Asia	302 (86.1%)	51 (26–74)	80.5%	84.1%	98.7%	78.5%	23.2%	18.2%	CD3^+^,CD4^+^, CD8^+^,Foxp3^+^	OS,DFS	8
Kobayashi 2007^35^	Asia	147 (76.9%)	62 (17–83)	38.1%	24.5%	92.5%	74.8%	NR	NR	CD8^+^, Foxp3^+^/CD4^+^	OS,DFS	6
Ikeguchi 2005^36^	Asia	59 (79.7%)	61.4 (28–80)	39%	NR	NR	NR	NR	57.6%	CD8^+^	OS	5
Ikeguchi 2004^37^	Asia	60 (80%)	61.6 ± 10.5	40%	60%	NR	NR	NR	NR	CD8^+^	DFS	7

NR: not reported.

^a^This study was divided into two independent terms, training cohort and validation cohort.

### Overall meta-analysis

#### CD8^+^ T lymphocytes

**Figure 1:**
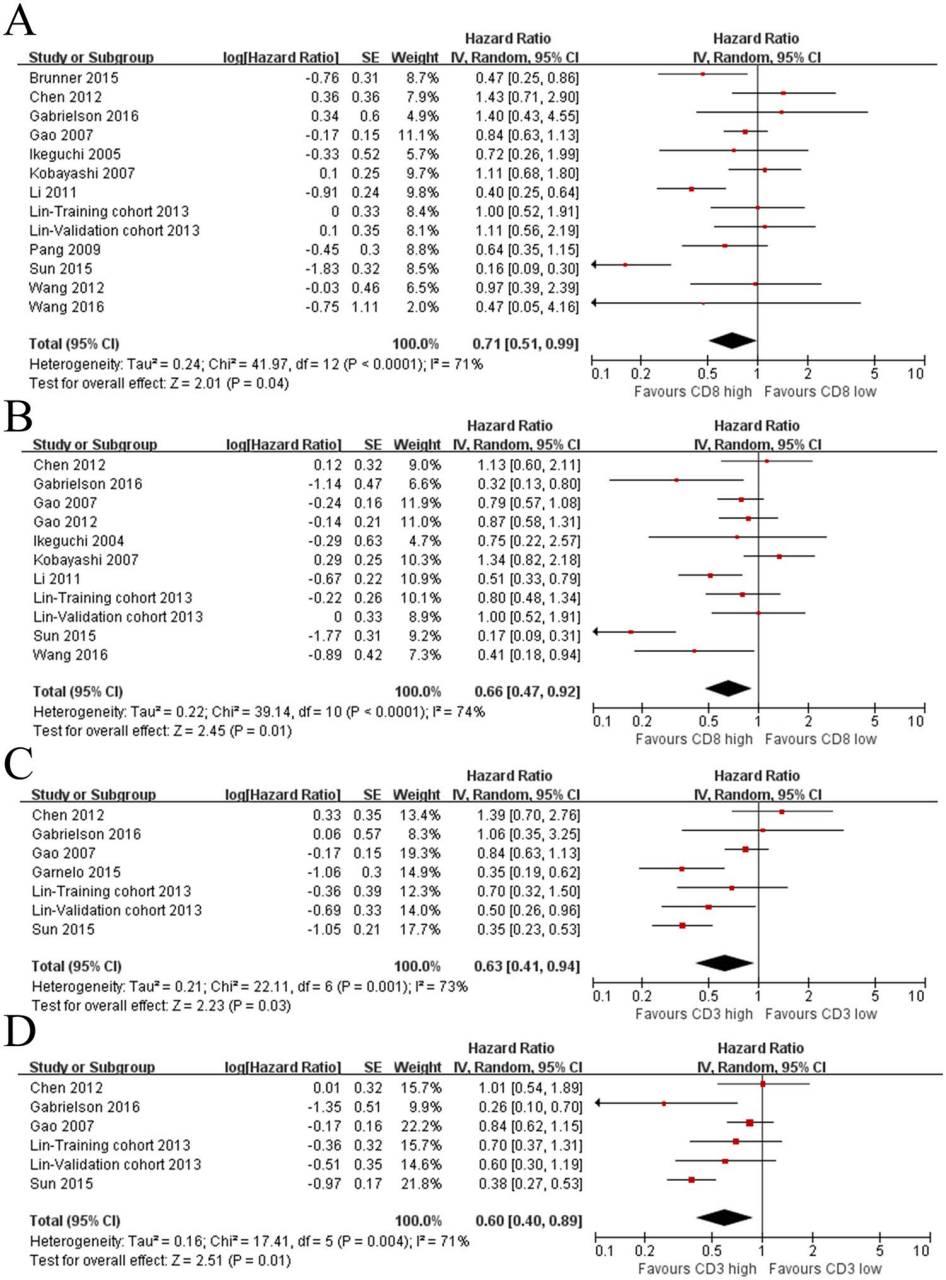
Forest plots of relationships between levels of CD8^+^ and CD3^+^ T lymphocytes and survival. (**A**) The effect of CD8^+^ T lymphocytes on OS. (**B**) The effect of CD8^+^ T lymphocytes on DFS/RFS. (**C**) The effect of CD3^+^ T lymphocytes on OS. (**D**) The effect of CD3^+^ T lymphocytes on DFS/RFS.

Fourteen studies of 2015 patients assessed the effects of CD8^+^ T lymphocytes on survival and were included in this meta-analysis. We concluded that a positive relationship existed between high levels of CD8^+^ T lymphocytes and OS (HR = 0.71; 95% CI, 0.51–0.99; *P* = 0.04; Fig. 1A). The effect on DFS/RFS was also significant (HR = 0.66; 95% CI, 0.47–0.92; *P* = 0.01; Fig. 1B).

We performed subgroup analyses to assess whether various clinical variables affected survival results (Supplementary Table S1). Patients with high levels of CD8^+^ T lymphocytes exhibited good OS based on the number of patients (<200; HR = 0.77; 95% CI, 0.62-0.94), percentage of HBsAg(+) patients (≥90%; HR = 0.26; 95% CI, 0.11–0.64), and percentage of patients with multiple tumors (<30%; HR = 0.39; 95% CI, 0.16–0.98). Patients with high levels of CD8^+^ T lymphocytes benefited from increased DFS/RFS based on the percentage of males (≥80%; HR = 0.64; 95% CI, 0.45–0.91), percentage of patients with liver cirrhosis (≥80%; HR = 0.77; 95% CI, 0.61–0.88), percentage of patients with multiple tumors (<30%; HR = 0.43; 95% CI, 0.19–0.93), and the percentage of vascular invasion (<50%; HR = 0.70; 95% CI, 0.57–0.86). Subgroup analyses indicated that CD8^+^ T lymphocytes were a positive predictor of OS or DFS/RFS in studies with these clinical variables.

### CD3^+^ T lymphocytes

Six studies involving 1421 patients detected the relationship between the infiltration of CD3^+^ T lymphocytes and HCC patient survival. The results indicated that high levels of CD3^+^ T lymphocytes were associated with good OS (HR = 0.63; 95% CI, 0.41–0.94; *P* = 0.03; Fig. 1C) and DFS/RFS (HR = 0.60; 95% CI, 0.40–0.89; *P* = 0.01; Fig. 1D).

Subgroup analyses were used to assess whether different clinical characteristics affected survival results. Supplementary Table S2 shows that patients with high levels of CD3^+^ T lymphocytes benefitted from increased OS based on the percentage of HBsAg(+) patients (≥90%; HR = 0.35; 95% CI, 0.23–0.53), percentage of patients with liver cirrhosis (≥80%; HR = 0.76; 95% CI, 0.59–0.98), percentage of Child-Pugh score A patients (<80%; HR = 0.58; 95% CI, 0.35–0.94), and percentage of vascular invasion (<50%; HR = 0.78; 95% CI, 0.61–0.99). The same results were noted for DFS/RFS based on the number of patients included in the study (<200; HR = 0.67; 95% CI, 0.47–0.95), the percentage of males (≥80%; HR = 0.66; 95% CI, 0.44–0.98; <80%; HR = 0.26; 95% CI, 0.10–0.71), the percentage of HBsAg(+) patients (≥90%; HR = 0.38; 95% CI, 0.27–0.53; <90%; HR = 0.76; 95% CI, 0.60–0.96), the percentage of TNM stage I-II patients (<70%; HR = 0.60; 95% CI, 0.38–0.97), and the percentage of vascular invasion (<50%; HR = 0.73; 95% CI, 0.56–0.93). We concluded that high levels of CD3^+^ T lymphocytes were a positive prognostic marker of OS or DFS/RFS in studies with these clinical variables.

### CD4^+^ T lymphocytes

**Figure 2:**
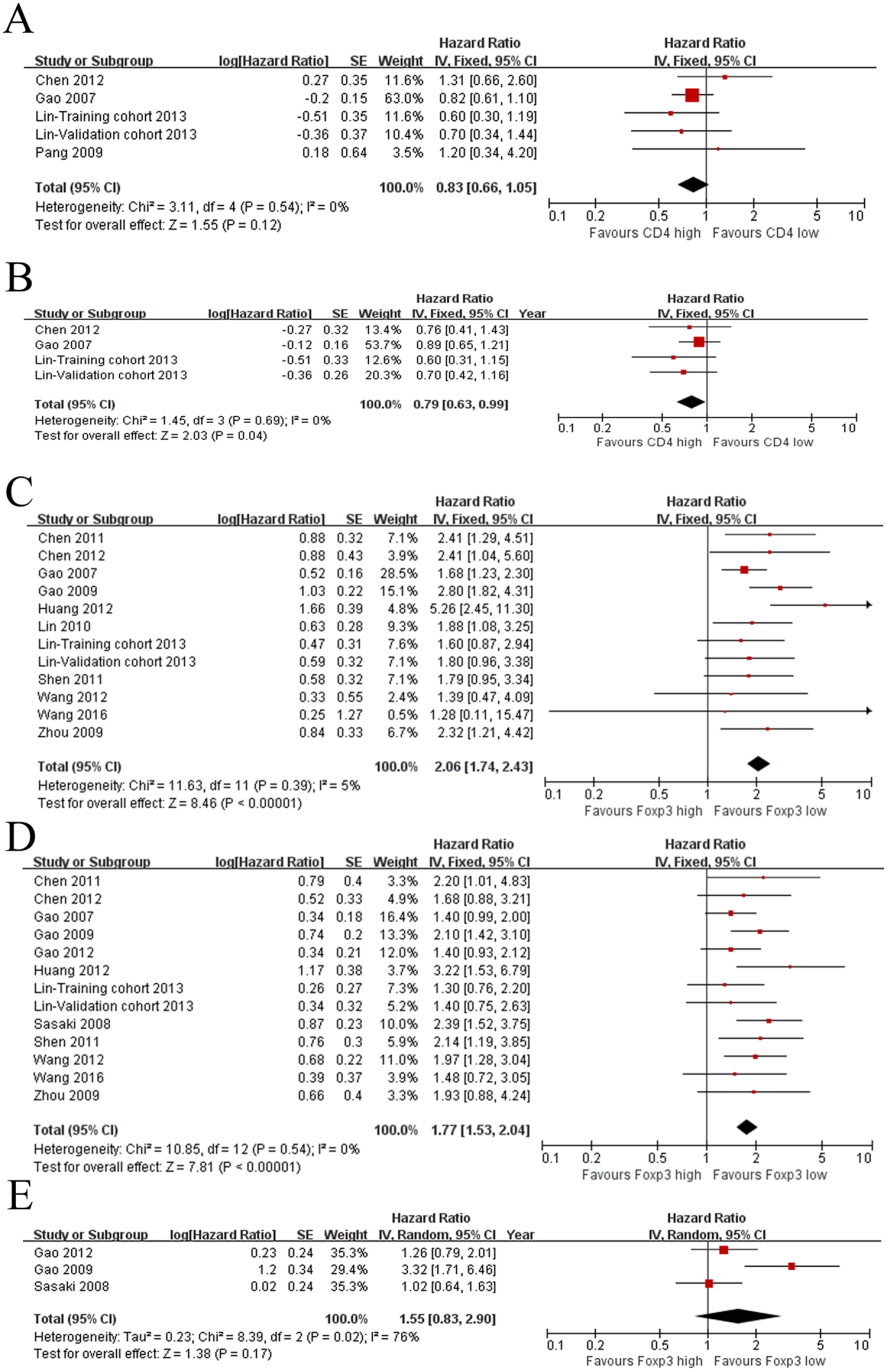
Forest plots of relationships between levels of CD4^+^ and Foxp3^+^ T lymphocytes and survival. (**A**) The effect of CD3^+^ T lymphocytes on OS. (**B**) The effect of CD3^+^ T lymphocytes on DFS/RFS. (**C**) The effect of Foxp3^+^ T lymphocytes on OS. (**D**) The effect of Foxp3^+^ T lymphocytes on DFS/RFS. (**E**) The effect of Foxp3^+^ T lymphocytes on CSS/CRS.

The prognostic value of CD4^+^ T lymphocytes was reported in four studies including 700 patients. High levels of CD4^+^ T lymphocytes exhibited no significant effect on OS (HR = 0.83; 95% CI, 0.66–1.05; *P* = 0.12; Fig. 2A), but improved DFS/RFS was observed (HR = 0.79; 95% CI, 0.63–0.99; *P* = 0.04; Fig. 2B).

Subgroup analyses were also used to assess the consistency of conclusions between different clinical characteristics of patients. Supplementary Table S3 shows that patients with high levels of CD4^+^ T lymphocytes exhibited improved DFS/RFS based on the number of patients included in the study (<200; HR = 0.69; 95% CI, 0.49–0.96), percentage of males (≥80%; HR = 0.79; 95% CI, 0.63–0.99), percentage of HBsAg(+) patients (<90%; HR = 0.79; 95% CI, 0.63–0.99), percentage of Child-Pugh score A patients (<80%; HR = 0.66; 95% CI, 0.44–0.98), percentage of TNM stage I-II patients (<70%; HR = 0.69; 95% CI, 0.49–0.96), and percentage of patients with multiple tumors (≥30%; HR = 0.69; 95% CI, 0.49–0.96). Subgroup analyses revealed that CD4^+^ T lymphocytes were a positive predictor of DFS/RFS in studies with these clinical variables.

### Foxp3^+^ Treg lymphocytes

Thirteen studies including 1961 patients reported the relationship between Foxp3^+^ T lymphocytes infiltration and HCC patient survival. Low levels of Foxp3^+^ lymphocytes were associated with improved OS (HR = 2.06; 95% CI, 1.74–2.43; *P* < 0.00001; Fig. 2C) and DFS/RFS (HR = 1.77; 95% CI, 1.53–2.04; *P* < 0.00001; Fig. 2D). However, the effect on CSS/CRS was not significant (HR = 1.55; 95% CI, 0.83–2.90; *P* = 0.17; Fig. 2E).

We also used subgroup analyses to assess the consistency of conclusions between different clinical variables. Notably, patients with low Foxp3^+^ T lymphocytes levels exhibit good OS and DFS/RFS based on all of the clinical variables and characteristics listed in Supplementary Table S4. Subgroup analyses demonstrated that Foxp3^+^ T lymphocytes were a poor predictor of OS and DFS/RFS in studies regardless of the number of patients (≥200 vs. <200), percentage of HBsAg(+) patients (≥90% vs. <90%), percentage of patients with liver cirrhosis (≥80% vs. <80%), percentage of Child-Pugh score A patients (≥80% vs. <80%), percentage of TNM stage I-II patients (≥70% vs. <70%), percentage of patients with multiple tumors (≥30% vs. <30%), and percentage of vascular invasion (≥50% vs. <50%). These results suggest that Foxp3^+^ T lymphocytes may be a validated risk biomarker of survival.

### Ratios between different subsets

**Figure 3:**
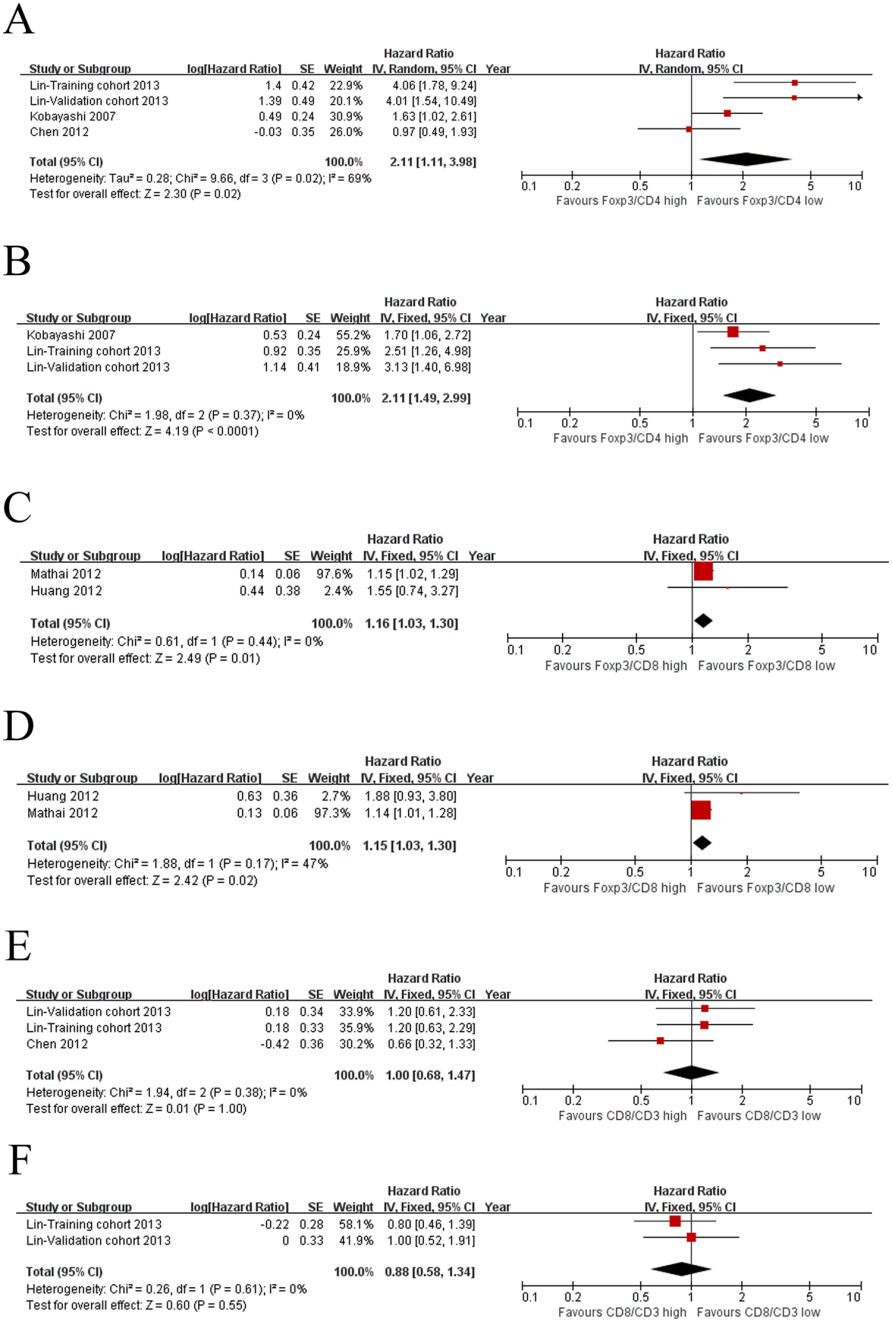
Forest plots of relationships between the Foxp3^+^/CD4^+^ ratio, Foxp3^+^/CD8^+^ ratio, CD8^+^/CD3^+^ ratio and survival. (**A**) The effect of the Foxp3^+^/CD4^+^ ratio on OS. (**B**) The effect of the Foxp3^+^/CD4^+^ ratio on DFS/RFS. (**C**) The effect of the Foxp3^+^/CD8^+^ ratio on OS. (**D**) The effect of the Foxp3^+^/CD8^+^ ratio on DFS/RFS. (**E**) The effect of the CD8^+^/CD3^+^ ratio on OS. (**F**) The effect of the CD8^+^/CD3^+^ ratio on DFS/RFS.

Different TIL subsets exhibited significant prognostic values on survival. Therefore, we continued to use a meta-analysis to evaluate the effect of ratios between different subsets on survival. There were 533 and 392 patients in the study of the effect of Foxp3^+^/CD4^+^ ratio on OS and RFS/DFS, respectively. Figure 3 shows that a low Foxp3^+^/CD4^+^ ratio correlated with improved OS (HR = 2.11; 95% CI, 1.11–3.98; *P* = 0.02; Fig. 3A) and DFS/RFS (HR = 2.11; 95% CI, 1.49–2.99; *P* < 0.0001; Fig. 3B). The effect of Foxp3^+^/CD8^+^ ratio on OS and DFS/RFS was analysed on 145 patients. A low Foxp3^+^/CD8^+^ ratio correlated with OS (HR = 1.16; 95% CI, 1.03–1.30; P = 0.01; Fig. 3C) and DFS/RFS (HR = 1.15; 95% CI, 1.03–1.30; P = 0.02; Fig. 3D). A total of 386 patients were included in a study on the effect of CD8^+^/CD3^+^ ratio on OS, and DFS/RFS was assessed in 245 patients. In contrast, the CD8^+^/CD3^+^ ratio exhibited no significant effect on OS (HR = 1.00; 95% CI, 0.68–1.47; P = 1.00; Fig. 3E) or DFS/RFS (HR = 0.88; 95% CI, 0.58–1.34; P = 0.55; Fig. 3F). These results indicate that the Foxp3^+^/CD4^+^ and Foxp3^+^/CD8^+^ ratios may be risk factors for survival.

### Publication bias

**Figure 4:**
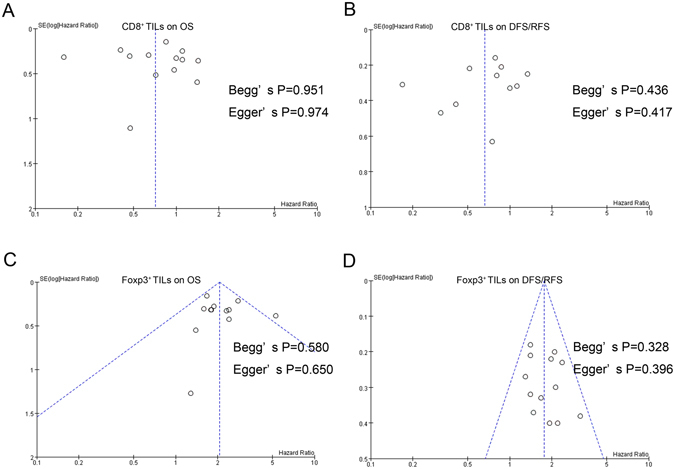
Funnel plots, Begg’s and Egger’s tests of the meta-analyses assessing the associations between TILs cells and survival. (**A**) Studies on the effect of CD8^+^ T cells on OS. (**B**) Studies on the effect of CD8^+^ T cells on DFS/RFS. (**C**) Studies on the effect of Foxp3^+^ T cells on OS. (**D**) Studies on the effect of Foxp3^+^ T cells on DFS/RFS.

Potential publication biases were evaluated by constructing a funnel plot and applying Begg’s test and Egger’s test. The results revealed that no publication bias existed in studies on CD8^+^ T lymphocytes in association with OS (Begg’s test, P = 0.951; Egger’s test, P = 0.974; Fig. 4A) and DFS/RFS (Begg’s test, P = 0.436; Egger’s test, P = 0.417; Fig. 4B). Similar results were noted for the effect of Foxp3^+^ TILs on OS (Begg’s test, P = 0.580; Egger’s test, P = 0.650; Fig. 4C) and DFS/RFS (Begg’s test, P = 0.328; Egger’s test, P = 0.396; Fig. 4D).

Regarding the sample sizes and studies about the the effect of CD3^+^ and CD4^+^ TILs on OS and DFS/RFS, Foxp3^+^ TILs on CRS/CSS were too little, we only conducted the Begg’s test and Egger’s test. The results provided no evidence of publication bias for CD3^+^ TILs on OS (Begg’s test, P = 0.548; Egger’s test, P = 0.995) and DFS/RFS (Begg’s test, P = 0.339; Egger’s test, P = 0.892), CD4^+^ TILs on OS (Begg’s test, P = 1.000; Egger’s test, P = 0.746) and DFS/RFS (Begg’s test, P = 0.308; Egger’s test, P = 0.110). We also found no publication bias for the Foxp3^+^ TILs on CRS/CSS (Begg’s test, P = 0.540; Egger’s test, P = 0.137).

## Discussion

Our increasing knowledge of the immune response and immune cells, especially tumor-infiltrating lymphocytes, support the significant value of these cells in multiple malignant tumors. Some studies assessed the prognostic value of tumor-infiltrating lymphocytes in various types of tumors, such as breast cancer, gastric cancer, non-small cell lung cancer, and ovarian cancer^38–41^. Many results indicated that TILs may be clinically significant prognostic biomarkers. A meta-analysis from MJM Gooden *et al*.^42^ reported the prognostic value of TILs in solid tumors. However, there were only 5 studies of HCC in this meta-analysis, and the prognostic data were analysed with various solid tumors that were not independently related to HCC. Some studies^43, 44^ individually reported the prognostic value of Foxp3^+^ T lymphocytes without reference to other subsets of TILs in HCC or were performed using the odds ratio (OR) rather than HR. It is almost impossible to perform perfect research on entire TILs subsets, and research to exclusively assess the prognostic value of Foxp3^+^ T lymphocytes may not represent the complete effect of TILs on survival. Further research should be performed to investigate the prognostic value of TILs in HCC. Our study strictly followed evidence-based medicine in this meta-analysis.

Our team performed a meta-analysis of 23 studies and 3173 patients using several authoritative databases. From these 23 articles involved in this meta-analysis, we could find some data show a strong relationship exist between TILs and survival, but others did not. These previous results made the effect of TILs on survival remain so controversial. However, through our systematic review and meta-analysis, we got a more unified conclusion that CD3^+^, CD4^+^, CD8^+^, and Foxp3^+^ could serve as prognostic biomarkers in hepatocellular carcinoma. We calculated HRs and 95% CIs associated with high versus low marker counts and demonstrated that high levels of CD8^+^ and CD3^+^ TILs improved OS, and high levels of CD8^+^, CD3^+^ and CD4^+^ TILs were associated with improved DFS/RFS. Therefore, these immune cells may be beneficial for survival. In contrast, Foxp3^+^ TILs levels and the Foxp3^+^/CD4^+^ and Foxp3^+^/CD8^+^ ratios were negatively associated with OS and DFS/RFS. Therefore, these factors may be prognostic risk factors for survival. Unfortunately, CD4^+^ TILs exhibited no statistical prognostic value on OS, and the CD8^+^/CD3^+^ ratio was not significantly related to OS or DFS/RFS. Sample size was too small to establish significant impacts of FoxP3+ TILs on CRS/CSS, we only found three studies and the heterogeneity was high (I2 = 76%). The current data are not comprehensive, and CRS/CSS was not generally reported. Therefore, further exploration is needed to obtain more credible data to analyse the effect of Foxp3^+^ TILs on CRS/CSS.

The mechanisms by which immune cells predict prognosis are not clear. Various types of immune cells play different roles in the tumor microenvironment, primarily via immunosuppressive and immunological effects. Some cells exert immunosuppressive effects, such as Foxp3^+^ Treg cells and mastocytes, and other cells exert immunological effects, such as cytotoxic T lymphocytes (CTLs), memory T lymphocytes, macrophages, and T helper lymphocytes. These effects are indispensable and influence each other. The determining factor of overall immune status depends on the sum of their effector functions or secretion of immuno-active substances. Immunological effector cells can be inhibited via the secretion of immunosuppressive factors, such as IL-10 and TGF-β1, granzyme and perforin expression, or competitive binding with IL-2 by immunosuppressive cells when the immunosuppressive role was strong, similar to the high levels of Foxp3^+^ TILs in this meta-analysis. These conditions promote the generation of immune tolerance and escape in tumor cells^45, 46^. These immune conditions hamper the anti-tumor immune response, which is more favourable for tumor growth and metastasis. Our results on the prognostic value of Foxp3^+^ TILs in this meta-analysis are consistent with this hypothesis and suggest that Foxp3^+^ TILs play pro-tumor roles. CD4^+^ and CD8^+^ TILs promote an immunoreaction against these extraneous agents in a manner similar to tumor cells and enhance anti-tumor immunity. This interaction may explain our conclusion of the prognostic value of CD8^+^ and CD4^+^ TILs. However, we did not completely reveal the complicated network connections, and the mechanisms of immune responses in oncology require further exploration.

Previous researchers investigated the effect of clinical characteristics on tumor prognosis. Information on patients’ clinical features is more visible and accessible for clinicians. Many researchers did not take this aspect into consideration. Our meta-analysis performed subgroup analyses of several clinical characteristics that were especially targeted to these HCC patients, such as hepatitis B virus (HBV) infection, liver cirrhosis, TNM stage, Child-Pugh score, and vascular invasion. Subgroup analyses were also crucial to discuss the sources of heterogeneity. The positive effect of CD8^+^ TILs on OS was associated with sample size, HBV infection, and tumor number. The positive effect on DFS/RFS was associated with sex, liver cirrhosis, tumor number, and vascular invasion. The positive effect of CD3^+^ TILs on OS was associated with HBV infection, liver cirrhosis, Child-Pugh score, and vascular invasion. The positive effect of CD3^+^ TILs on DFS/RFS was associated with sample size, sex, HBV infection, TNM stage, and vascular invasion. The effect of CD4^+^ TILs on OS was related to sample size, sex, HBV infection, Child-Pugh score, TNM-stage, and tumor number. The prognostic value of Foxp3^+^ TILs on OS or DFS/RFS was associated with all of the listed clinical characteristics, which demonstrated that Foxp3^+^ TILs was a valuable and impressive poor predictor of survival. These results suggest that clinicians should pay more attention to these clinical features on survival.

Several limitations of this meta-analysis exist despite the rigorous design. First, we could not create a unified standard to identify high or low levels of TILs or the ratios. Different standards were used in various studies, and concrete data of expression levels were not accessible. These defects prevented us from advancing more reliable results. Second, publication bias was not assessed for studies on CD3^+^ and CD4^+^ TILs because of the limited number of published studies, which may influence the applicability of the results. High heterogeneity was frequently noted, especially in studies on CD8^+^ and CD3^+^ TILs, despite our use of several subgroup analyses. Therefore, we urge researchers to perform studies derived from more homogeneous populations. Then, after scrutinizing these 23 articles, we found only one study was conducted with training cohort and testing cohort. So we should conduct further research with training cohort and testing cohort to set up more predictive value of TIL data. Finally, this meta-analysis was based on retrospective studies with some unavoidable deficiencies, such as insufficient information on alcoholism and smoking history, surgical methods and therapeutic approaches, and lymph nodes. These confounding variables may affect the prognostic results.

This meta-analysis demonstrated the prognostic value of TILs in HCC using a comprehensive literature search, data extraction, and outcomes measured despite these limitations. This study provides significant information on TIL subsets, such as CD3^+^, CD4^+^, CD8^+^, and Foxp3^+^, and indicates that they can be used as prognostic biomarkers for HCC or as targeted molecules for anti-tumor treatment. Our research advanced current knowledge of the functions of the immune responses in oncology. Future rigorous studies of the effect of TILs in cancer are encouraged to promote human health.

## Methods

### Literature search

Two independent reviewers identified relevant articles via an electronic search of PubMed, Embase, and Web of Science. The electronic database was searched from study inception to April 2017. The following terms were used: “Lymphocytes, Tumor-Infiltrating”, “TILs” “T Lymphocytes”, “FoxP3-positive T lymphocytes”, “CD8-Positive T Lymphocytes”, “CD3-Positive T Lymphocytes”, “CD4-Positive T Lymphocytes”, “prognosis”, “survival”, “recurrence free survival”, “disease free survival”, “Carcinomas, Hepatocellular”, “Hepatocellular Carcinoma” and “HCC”. Additional pertinent studies were incorporated from a review of the reference lists of selected papers and related articles.

### Inclusion and exclusion criteria

Eligible studies were assessed using the following criteria: (1) the prognostic value of CD3^+^, CD4^+^, CD8^+^, and FoxP3^+^ T lymphocytes as subsets of TILs were examined, including their ratios; (2) these lymphocyte markers were detected using immunohistochemistry from human tissues; (3) the related research should originate from original articles; (4) prognostic indicators were calculated as OS, DFS/RFS, or CSS/CRS; and (5) hazard ratios (HRs) and 95% confidence intervals (95% CIs) were used as effect measures or adequate data for calculating HRs and 95% CIs were provided, such as Kaplan–Meier curves.

The following exclusion criteria were used: (1) reviews, case reports, conference abstracts, editorials, and expert opinion; (2) non-English articles; (3) lymphocytes markers were detected in peri-tumoral tissues or peripheral blood; and (4) non-primary HCC, such as colorectal liver metastases. We used updated and proximate articles if similar data were repeated in several articles.

### Data extraction and outcome measure

Two independent reviewers extracted data based on the criteria mentioned above. Disagreements were resolved by consensus or a re-review of the article. The following data were extracted from articles: first author, year of publication, ethnicity, mean or median age_(year), number of patients and sex, TILs subsets, and outcomes measured. We also recorded relative information on HBV infection, liver cirrhosis, TNM-stage, Child-Pugh score, tumor number, and vascular invasion, especially for the percentage of patients with a certain kind of clinical characteristic in a independent study. In subgroup analyses, the research is divided into two groups of studies with different demographics for each clinical characteristic.

HRs and 95% CIs were used as effect measures. Univariate and multivariate analyses were performed, and we selected the latter analysis for more accurate HRs and 95% CIs. If Kaplan–Meier curves were available rather than HRs, then we calculated HRs using the tabulation from Tierney *et al*.^47^, which is based on the method reported by Parmar *et al*.^48^. HRs and 95% CIs for survival were associated with high versus low levels of TILs. Therefore, when the data were associated with low levels versus high levels of TILs, the reciprocals of HRs and 95% CIs were calculated to indicate the effect on survival. The weighting was used to mean the proportion of each study’s result in the overall results. The weighting depends on the samples size and estimated value of effects in this study. The larger the sample size is, or more accurate the estimates value of effect is, the bigger the weighting is.

We have used the Newcastle-Ottawa Scale (NOS) to assess the quality of studies with its design, content and ease of use directed to the task of incorporating the quality assessments in the interpretation of meta-analytic results^49^. NOS scores of the studies ranged from 5 to 8, which were considered high quality.

### Statistical analysis

We used HRs and their 95% CIs to demonstrate the relationship between TILs and patients prognosis, including their ratios. Furthermore, HRs less than 1 represented a better survival result for patients with high levels of TILs based on the data estimated with high versus low levels of TILs. *P* < 0.05 indicated statistically significant results. The *χ*2 test and *I* ^2^ index were used to measure the heterogeneity^50^, which may represent the degree of heterogeneity resulting from variables between studies (25% low heterogeneity, 50% medium, 75% high). A fixed-effect model was used only when *I* ^2^ < 50% and *P* > 0.1. Otherwise, we used a random-effects model. Subgroup analyses were performed when the overall results had statistical significance to investigate potential sources of heterogeneity and assess whether various clinical variables or study characteristics affected survival results. We also used funnel plots and Begg’s and Egger’s tests^51, 52^ to detect publication bias. *P* < 0.05 indicated publication bias, and *P* > 0.05 indicated no bias. All statistical analyses were performed with Revman software (version 5.3; Cochrane Collaboration, Oxford, United Kingdom), with the exception of the Begg’s and Egger’s tests, which were assessed using STATA12.0.

## Electronic supplementary material

Supplementary Tables
