# Supplementary material for: The Prognostic Value of Tumor-infiltrating Lymphocytes in Hepatocellular Carcinoma: a Systematic Review and Meta-analysis
Source: Sci Rep. 2017 Aug 8;7:7525. doi: 10.1038/s41598-017-08128-1 (PMC5548736; doi:10.1038/s41598-017-08128-1)
Supplement: Supplementary file 1 — Supplementary Tables [file 41598_2017_8128_MOESM1_ESM.pdf]

The Prognostic Value of Tumor-infiltrating Lymphocytes in Hepatocellular Carcinoma: a Systematic Review and Meta-analysis

Wei Yao[1#], Jun-chuang He[2#], Yan Yang[3], Jian-ming Wang[1*], Ya-wei Qian[1], Tao Yang[1], Lei Ji[1]

Supplementary Table S1. Subgroup analyses for $CD8^+$ T lymphocytes

	Studies(*n*)	Patients(*n*)	HR(95%CI)	*P*-value	I^2	*P*(heterogeneity)
**$CD8^+$(OS)**						
**No.of patients,≥200**	2	661	0.38(0.07-1.92)	0.24	95%	<0.00001
**No.of patients,<200**	10	1188	0.77(0.62-0.94)	0.01	46%	0.05
**Male, ≥80%**	8	1459	0.67(0.43-1.04)	0.07	77%	<0.0001
**Male,<80%**	4	390	0.82(0.58-1.15)	0.25	46%	0.13
**HBsAg(+),≥90%**	2	556	0.26(0.11-0.64)	0.003	81%	0.02
**HBsAg(+),<90%**	8	1215	0.91(0.75-1.09)	0.30	2%	0.41
**Liver cirrhosis,≥80%**	5	947	0.77(0.61-1.09)	0.14	49%	0.08
**Liver cirrhosis,<80%**	5	831	0.68(0.29-1.56)	0.36	87%	<0.00001
**Child-pugh A, ≥80%**	4	787	0.83(0.51-1.33)	0.44	76%	0.006
**Child-pugh A,<80%**	3	501	0.80(0.57-1.13)	0.20	33%	0.21
**TNM stage** I-II, **≥70%**	4	711	0.78(0.42-1.27)	0.32	72%	0.01
**TNM stage** I-II,**<70%**	6	942	0.63(0.35-1.15)	0.13	79%	<0.0001
**Multiple tumor,≥30%**	3	523	1.12(0.78-1.61)	0.53	0	0.88
**Multiple tumor,<30%**	3	858	0.39(0.16-0.98)	0.04	92%	<0.00001
**Vascular invasion,≥50%**	3	266	1.08(0.62-1.89)	0.79	0	0.41
**Vascular invasion,<50%**	4	809	0.80(0.53-1.20)	0.28	61%	0.03
**$CD8^+$(DFS/RFS)**						
**No.of patients,≥200**	3	867	0.51(0.22-1.15)	0.11	91%	<0.0001
**No.of patients,<200**	7	921	0.74(0.53-1.05)	0.09	57%	0.02
**Male, ≥80%**	8	1576	0.64(0.45-0.91)	0.01	73%	0.0003
**Male,<80%**	2	212	0.69（0.17-2.79)	0.60	86%	0.007
**HBsAg(+),≥90%**	3	762	0.43（0.19-1.01)	0.05	89%	<0.0001
**HBsAg(+),<90%**	6	960	0.88（0.72-1.07)	0.19	32%	0.18
**Liver cirrhosis,≥80%**	5	1016	0.77（0.61-0.88)	0.001	22%	0.27
**Liver cirrhosis,<80%**	5	772	0.58（0.23-1.41)	0.23	88%	<0.00001

**Child-pugh A, ≥80%**	4	787	0.86（0.57-1.28）	0.45	68%	0.02
**Child-pugh A,<80%**	2	451	0.87（0.65-1.16）	0.35	0	0.87
**TNM stage** I-II, **≥70%**	4	711	0.70（0.43-1.13）	0.14	74%	0.008
**TNM stage** I-II,**<70%**	5	1017	0.63（0.36-1.09）	0.10	81%	<0.0001
**Multiple tumor,≥30%**	3	592	0.91（0.70-1.18）	0.48	0	0.85
**Multiple tumor,<30%**	3	858	0.43（0.19-0.93）	0.03	90%	<0.0001
**Vascular invasion,≥50%**	3	413	0.83（0.61-1.14）	0.25	47%	0.15
**Vascular invasion,<50%**	4	809	0.70（0.57-0.86）	0.0008	41%	0.15

Supplementary Table S2. Subgroup analyses for $CD3^+$ T lymphocytes

	Studies(*n*)	Patients(*n*)	HR(95%CI)	*P*-value	I^2	*P*(heterogeneity)
**$CD3^+$(OS)**						
**No.of patients,≥200**	2	661	0.55(0.23-1.30)	0.17	91%	0.0006
**No.of patients,<200**	4	554	0.67(0.40-1.14)	0.14	62%	0.03
**Male, ≥80%**	4	1020	0.66(0.41-1.07)	0.09	77%	0.002
**Male,<80%**	1	65	1.06	0.92	NA	NA
**HBsAg(+),≥ 90%**	1	359	0.35(0.23-0.53)	<0.00001	NA	NA
**HBsAg(+),<90%**	4	753	0.83(0.62-1.10)	0.20	18%	0.30
**Liver cirrhosis,≥80%**	2	547	0.76(0.59-0.98)	0.04	6%	0.35
**Liver cirrhosis,<80%**	3	538	0.76(0.27-2.12)	0.60	85%	0.002
**Child-pugh A, ≥80%**	2	443	0.91(0.70-1.19)	0.50	42%	0.19
**Child-pugh A,<80%**	1	245	0.58(0.35-0.94)	0.03	NA	NA
**TNM stage** I-II, **≥70%**	2	367	0.86(0.64-1.14)	0.28	0	0.70
**TNM stage** I-II,**<70%**	3	745	0.62(0.34-1.14)	0.12	75%	0.007
**Multiple tumor,≥30%**	2	386	0.78(0.43-1.44)	0.43	57%	0.1
**Multiple tumor,<30%**	2	661	0.55(0.23-1.30)	0.17	91%	0.0006
**Vascular invasion,≥50%**	1	141	1.39(0.70-2.76)	0.35	NA	NA
**Vascular invasion,<50%**	3	612	0.78(0.61-0.99)	0.04	0	0.49
**$CD3^+$(DFS/RFS)**						
**No.of patients,≥200**	2	661	0.57(0.26-1.24)	0.16	91%	0.0006
**No.of patients,<200**	3	451	0.67(0.47-0.95)	0.02	43%	0.16
**Male, ≥80%**	4	1047	0.66(0.44-0.98)	0.04	73%	0.006
**Male,<80%**	1	65	0.26(0.10-0.71)	0.008	NA	NA
**HBsAg(+),≥ 90%**	1	359	0.38(0.27-0.53)	<0.00001	NA	NA
**HBsAg(+),<90%**	4	753	0.76(0.60-0.96)	0.02	35%	0.19
**Liver cirrhosis,≥80%**	2	547	0.78(0.60-1.01)	0.06	0	0.63
**Liver cirrhosis,<80%**	3	565	0.48(0.23-1.01)	0.05	77%	0.01
**Child-pugh A, ≥80%**	2	443	0.87(0.66-1.16)	0.35	0	0.61
**Child-pugh A,<80%**	1	245	0.65(0.41-1.04)	0.07	0	0.75
**TNM stage** I-II, **≥70%**	2	302	0.57(0.26-1.24)	0.16	91%	0.0006
**TNM stage** I-II,**<70%**	3	745	0.60(0.38-0.97)	0.04	66%	0.03
**Multiple tumor,≥30%**	2	386	0.76(0.52-1.10)	0.15	0	0.52
**Multiple tumor,<30%**	2	661	0.57(0.26-1.24)	0.16	91%	0.0006
**Vascular invasion,≥50%**	1	141	1.01(0.54-1.89)	0.98	NA	NA
**Vascular invasion,<50%**	3	612	0.73(0.56-0.93)	0.01	43%	0.15

NA: not applicable

Supplementary Table S3. Subgroup analyses for $CD4^+$ T lymphocytes

	**Studies(*n*)**	**Patients(*n*)**	**HR(95%CI)**	***P*-value**	***I*²**	***P*(heterogeneity)**
**$CD4^+$(DFS/RFS)**						
**No.of patients,≥200**	1	302	0.89(0.65-1.21)	0.45	NA	NA
**No.of patients,<200**	2	386	0.69(0.49-0.96)	0.03	0	0.87
**Male, ≥80%**	3	688	0.79(0.63-0.99)	0.04	0	0.69
**Male,<80%**	0	NA	NA	NA	NA	NA
**HBsAg(+),≥ 90%**	0	NA	NA	NA	NA	NA
**HBsAg(+),<90%**	3	688	0.79(0.63-0.99)	0.04	0	0.69
**Liver cirrhosis,≥80%**	2	547	0.79(0.62-1.01)	0.06	0	0.49
**Liver cirrhosis,<80%**	1	141	0.76(0.41-1.43)	0.40	NA	NA
**Child-pugh A, ≥80%**	2	443	0.86(0.65-1.14)	0.29	0	0.68
**Child-pugh A,<80%**	1	245	0.66(0.44-0.98)	0.04	NA	NA
**TNM stage** I-II, **≥70%**	1	302	0.89(0.65-1.21)	0.45	NA	NA
**TNM stage** I-II,**<70%**	2	386	0.69(0.49-0.96)	0.03	0	0.87
**Multiple tumor,≥30%**	2	386	0.69(0.49-0.96)	0.03	0	0.87
**Multiple tumor,<30%**	1	302	0.89(0.65-1.21)	0.45	NA	NA
**Vascular invasion,≥50%**	1	141	0.76(0.41-1.43)	0.40	NA	NA
**Vascular invasion,<50%**	2	547	0.79(0.62-1.01)	0.06	0	0.49

Supplementary Table S4. Subgroup analyses for Foxp3^{+} T lymphocytes

	Studies(*n*)	Patients(*n*)	HR(95%CI)	*P*-value	I^2	*P*(heterogeneity)
**Foxp3^{+}(OS)**						
**No.of patients,≥200**	2	542	2.12(1.29-3.49)	0.003	72%	0.06
**No.of patients,<200**	9	1049	2.10(1.68-2.62)	<0.00001	0	0.53
**HBsAg(+),≥ 90%**	2	316	2.41(1.69-3.42)	<0.00001	29%	0.24
**HBsAg(+),<90%**	6	1022	1.95(1.58-2.42)	<0.00001	32%	0.18
**Liver cirrhosis,≥80%**	7	1120	2.03(1.68-2.46)	<0.00001	28%	0.19
**Liver cirrhosis,<80%**	3	386	2.15(1.48-3.12)	<0.0001	0	0.81
**Child-pugh A, ≥80%**	5	902	2.04(1.65-2.53)	<0.00001	3%	0.39
**Child-pugh A,<80%**	3	436	2.17(1.23-3.83)	0.008	58%	0.07
**TNM stage** I-II, **≥70%**	3	644	1.98(1.58-2.50)	<0.00001	44%	0.17
**TNM stage** I-II,**<70%**	5	649	2.28(1.69-3.08)	<0.00001	25%	0.25
**Multiple tumor,≥30%**	4	666	1.91(1.40-2.62)	<0.0001	0	0.82
**Multiple tumor,<30%**	4	672	2.41(1.55-3.73)	<0.0001	68%	0.02
**Vascular invasion,≥50%**	3	261	3.51(2.02-6.10)	<0.00001	19%	0.29
**Vascular invasion,<50%**	3	787	1.92(1.54-2.39)	<0.00001	25%	0.26
**Foxp3^{+}(DFS/RFS)**						
**No.of patients,≥200**	3	748	1.60(1.28-1.99)	<0.0001	27%	0.26
**No.of patients,<200**	9	1111	1.90(1.58-2.29)	<0.00001	0	0.67
**Male, ≥80%**	10	1610	1.70(1.46-1.98)	<0.00001	0	0.55
**Male,<80%**	1	164	2.39(1.52-3.75)	0.0002	NA	NA
**HBsAg(+),≥ 90%**	3	522	1.51(1.18-1.93)	0.0009	0	0.44
**HBsAg(+),<90%**	7	1186	1.95(1.62-2.35)	<0.00001	0	0.51
**Liver cirrhosis,≥80%**	8	1326	1.68(1.43-1.98)	<0.00001	5%	0.39
**Liver cirrhosis,<80%**	3	448	2.14(1.53-2.99)	<0.00001	0	0.68
**Child-pugh A, ≥80%**	5	902	1.77(1.43-2.20)	<0.00001	0	0.54
**Child-pugh A,<80%**	4	642	1.65(1.31-2.07)	<0.0001	26%	0.25
**TNM stage** I-II, **≥70%**	2	542	1.70(1.15-2.51)	0.008	55%	0.14
**TNM stage** I-II,**<70%**	5	712	1.55(1.22-1.96)	0.0003	0	0.48
**Multiple tumor,≥30%**	5	872	1.59(1.28-1.98)	<0.0001	0	0.74
**Multiple tumor,<30%**	4	672	2.02(1.47-2.79)	<0.0001	50%	0.11
**Vascular invasion,≥50%**	4	467	1.66(1.25-2.22)	0.0006	21%	0.29
**Vascular invasion,<50%**	3	787	1.57(1.26-1.96)	<0.0001	3%	0.38

NA: not applicable
